# Diagnostic accuracy of CD44V6 for osteosarcoma: a meta-analysis

**DOI:** 10.1186/s13018-016-0470-2

**Published:** 2016-11-03

**Authors:** Yunyuan Zhang, Limin Lun, Baozhi Zhu, Qing Wang, Chunming Ding, Yanlin Hu, Weili Huang, Lan Zhou, Xian Chen, Hai Huang

**Affiliations:** 1 Department of Clinical Laboratory, The Affiliated Hospital of Qingdao University, Qingdao, 266003 People’s Republic of China; 2 Department of Orthopedic Surgery, Qingdao Municipal Hospital, Qingdao, 266011 People’s Republic of China; 3 Department of Trauma Surgery, The Affiliated Hospital of Qingdao University, Qingdao, 266003 People’s Republic of China; 4 Department of Infection Control, The Affiliated Hospital of Qingdao University, Qingdao, 266003 People’s Republic of China; 5 College of Laboratory Medicine, Key Laboratory of Laboratory Medical Diagnostics designated by Chinese Ministry of Education, Chongqing Medical University, Chongqing, 400016 China; 6 Department of Clinical Biochemistry, School of Clinical Laboratory Science, Guizhou Medical University, Guiyang, 550004 Guizhou Province China; 7 Department of Clinical Biochemistry, Affiliated Hospital of Guizhou Medical University, Guiyang, 550004 Guizhou Province China

**Keywords:** CD44V6, Osteosarcoma, Diagnosis, Meta-analysis

## Abstract

**Background:**

Recently, more and more evidences have revealed the association between CD44V6 and osteosarcoma (OS), but whether it can be used as a clinical biomarker is still unknown. The purpose of this study is to assess the diagnostic value of CD44V6 in OS by conducting a meta-analysis.

**Methods:**

All relevant electronic literatures were collected from seven international databases together with three Chinese databases up to April 23, 2016. Eligible studies were selected through multiple search strategies and the quality was assessed by QUADAS. Data was extracted from studies according to the key statistics index. All analyses were performed using STATA 12 and Meta-DiSc 1.4 statistical software.

**Results:**

According to the exclusion and inclusion criteria, 8 literatures were retrieved, accounting for 463 cases and 188 controls. For discriminating OS from benign bone tumor or healthy controls, the area under the receiver operating characteristic curve (AUC) was 0.91 (95 % CI 0.88–0.93). Overall, the results showed pooled sensitivity of 0.743 (95 % CI 0.606–0.844) and specificity of 0.897 (95 % CI 0.818–0.945), respectively. Substantial heterogeneity was detected in this study (*I* ^2^ = 90 %). The publication bias was assessed by using Deeks’ asymmetry test (*p* = 0.795). No evidence of heterogeneity from threshold effects was detected by the Spearman correlation coefficient (−0.506, *p* = 0.201). Meta-regression was performed to mining the source of heterogeneity, and subgroup analysis showed that neither the cut-off values nor the control groups were the source of heterogeneity.

**Conclusions:**

The present results suggest that promoted CD44V6 expression levels are associated with OS and CD44V6 may be used as a diagnostic marker for OS.

## Background

Osteosarcoma (OS) is the most frequent primary non-hematological bone tumor that mainly afflicts adolescents, and the peak incidence is during the second decade of life [1, 2]. Proximal tibia, proximal humerus, and distal femur are the most common afflicted sites of primary tumors [3]. The majority of OS patients have high grade lesions and poor prognosis. As the second leading cause of cancer associated death in young adults, approximately 80 % of OS patients have metastatic disease at the time of diagnosis [4]. Although plain radiographic imaging is highly suggestive for OS diagnosis, only 10–15 % of these lesions are detectable with current diagnostic tools [5].

Currently, the mechanism of oncogenesis and tumor progression is still not fully elucidated, and this restricts the diagnosis of OS. Many scientists endeavored to OS diagnosis, prognosis, and treatment because effective diagnostic biomarker and therapeutic methods used for OS have not been discovered. Laboratory evaluation for OS patients is generally normal, and the serum alkaline phosphatase (ALP), phosphatase lactate dehydrogenase, and MMP9 levels have been documented, unspecified, and up-regulated in 40–50 % of patients [6]. After caner resection, the levels of serum ALP are generally decreased, but it is strongly suggested the recurrence and metastasis when declined ALP values are elevated again [7]. Some researchers had suggested that identifying ideal diagnostic markers in cancer would be valuable for proper individual management. Therefore, it would be urgently needed to explore more sensitive and specific non-invasive biological biomarkers for early OS diagnosis.

As a trans-membrane glycoprotein, CD44 has a cytoplasmic domain, a trans-membrane domain, and seven extracellular domains [8]. CD44 variant isoform V6 (CD44V6) is one of the variant isoforms (CD44V), which is reportedly associated with increased invasion, metastasis, and poor prognosis of different neoplasms [9–12]. CD44V6 not only regulates the extracellular matrix and promotes cell motility but also suppresses tumor apoptosis and promotes tumor progression. Although some studies showing that CD44V6 confer a pivotal diagnostic value in various solid tumors, the association between CD44V6 and OS were still controversial. In order to further validate the clinical applicability of CD44V6 for OS, we conducted this systematic meta-analysis based on all relevant studies.

## Methods

### Search strategy and selection criteria

The Cochrane Library, PubMed (MEDLINE), ISI Web of Knowledge, ScienceDirect, Embase, BioMed Central, and Springer together with three Chinese databases Weipu, Wanfang, and China National Knowledge Internet (CNKI) databases were used to conduct a comprehensive computerized literature search for articles that evaluated the accuracy of CD44V6 for the diagnosis of OS. The studies were identified by using the following keywords in variably combinations: “(osteosarcoma OR bone tumor) and (CD44V6 OR CD44 variation 6).” In addition to the electronic literatures that published before April 23, 2016, the reference lists of primary studies and previous systematic reviews were also searched for additional articles.

### Quality assessment

The Quality Assessment of Diagnostic Accuracy Studies checklist (QUADAS) were used to assess the methodological quality of the studies [13]. The guidelines for scoring each item to our analysis were tailored [14]. In summary, the involved articles were considered low risk of bias according to the QUADAS criteria. Review protocol can be accessed on the site http://www.crd.york.ac.uk/PROSPERO/ with registration number CRD42016037459.

### Study selection criteria

Inclusion criteria are as follows: (1) measurement of CD44V6 in OS using commercial reagents; (2) definite diagnosis confirmed for newly diagnosed patients with OS as the case group and patients with benign bone tumor (BBD) or healthy people as the control group; (3) studies with sufficient information to construct the 2 × 2 contingency table; (4) publications written in English or Chinese.

Exclusion criteria are as follows: (1) literatures not pertinent to CD44V6; OS diagnosed without a biopsy and there was no clear cut-off value in the literature; (2) no control groups or control group is not BBD or healthy people; (3) similar studies from the same author as well as multiple duplicate data in the different works, excluding earlier and smaller sample data; (4) animal experiments, case reports, correspondences, reviews, expert opinions, letters, talks, and editorials without original data.

### Data extraction

Data was carefully extracted from all eligible studies in duplicate by two independent investigators (YYZ and XC). Extracted databases were crosschecked between the two authors to rule out any discrepancy. Disagreement was resolved by consulting with a third investigator. The following data for each collected studies were extracted independently: (1) basic information of articles (the first author, publication year); (2) characterization of research objects (sample size, assay kit, cut-off value); and (3) data used to calculate the sensitivity and specificity of each study (TP, FP, FN, TN). If any essential information were not available from the article, best efforts were made to sending a reminder to the corresponding authors. The study was excluded if no response was received after sending a reminder.

### Statistical analysis

Stata 12.0 (Stata Corporation, College Station, TX, USA) and Meta-DiSc 1.4 software were used for all statistical analyses. The true positives, false negatives, false positives, and true negatives in each study were tabulated to obtain pooled sensitivity (SEN), pooled specificity (SPE), positive likelihood ratio (PLR), negative likelihood ratio (NLR) and a corresponding CI. Summary receiver operating characteristic curve (SROC) was used to summarize the results [15, 16]. The respective area under the SROC curve (AUC) and Q point value (Q) were estimated to evaluate the accuracy of the diagnostic test [16]. Generally, the score of AUC 0.93 to 0.96 is regarded as very good, and 0.75–0.92 as good, but AUC <0.75 can be still reasonable [17]. If heterogeneity among studies was recorded by *I* ^2^, the potential source of heterogeneity was investigated by sensitivity analysis and meta-regression. Study specific covariates such as cut-off values, control groups and assay kits were used for investigated the meta-regression reason. Deeks’ regression test and Spearman’s correlation coefficient (rs) of log (SEN) and log (1-SPE) were used to inspect publication bias.

## Results

### Study characteristics

**Fig. 1:**
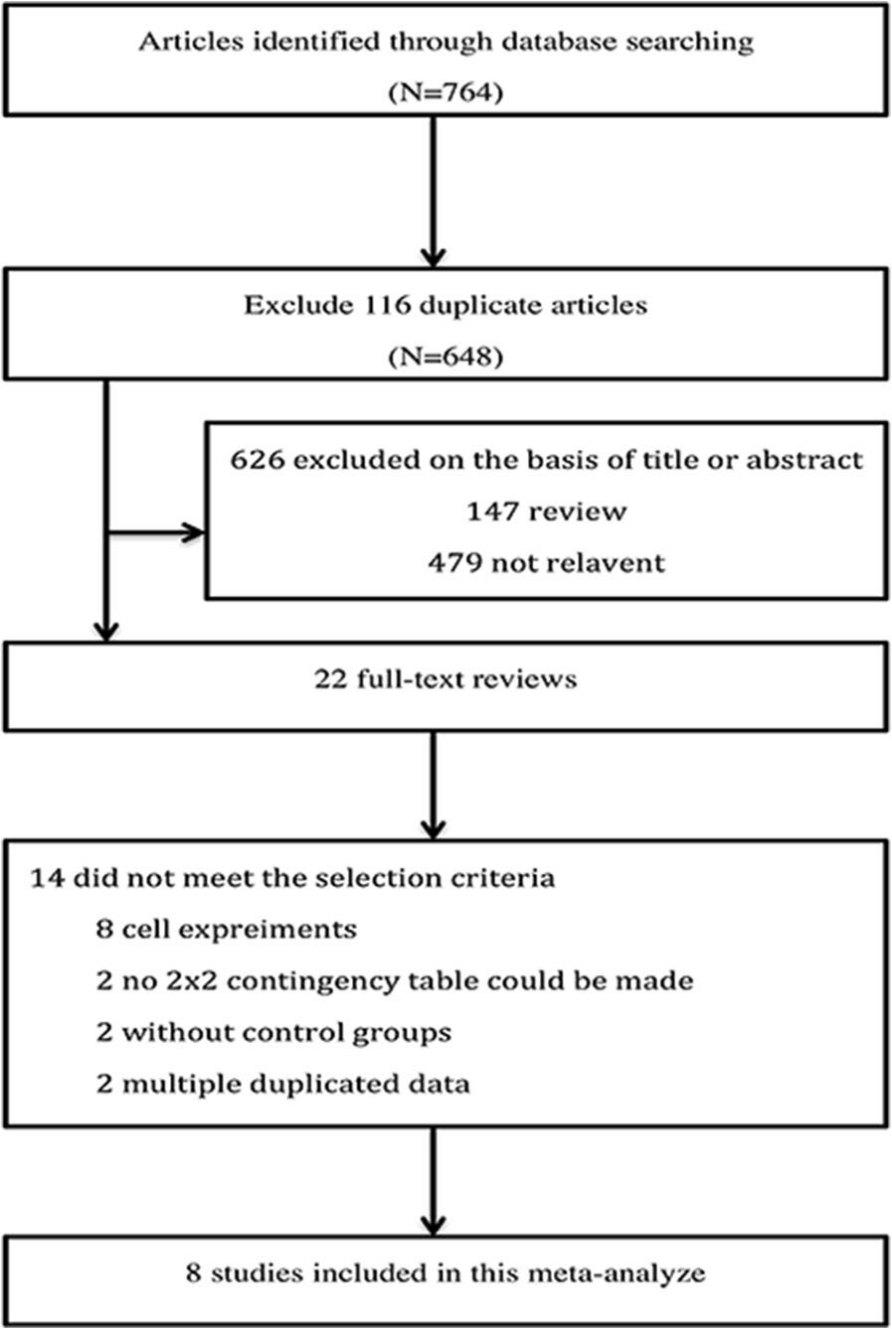
Schematic representation of the study selection

As a result of electronic databases search, 764 published records associated with the diagnostic value of CD44V6 in OS were retrieved. 648 articles were left after duplicated data removed. After reviewing the titles and abstracts, we excluded another 626. After a full text review we excluded a further 14, finally, 8 studies [18–25] were included in our study (Fig. 1). As shown in Table 1, the expression level of CD44V6 was detected by immunohistochemistry (IHC) in all studies and the results were judged by cut-off value in 2 ways: immunoreactivity score (IRS) or positivity percentage. According to Enneking System, 4 of 8 articles reported classification of clinical stages [26] (stages 1, 2, and 3; Table 2).

**Table 1:** Main characteristics of 8 studies

Author	Year	Assay kit	*n*	Case of OS	Case of control		Prevalence (%)	Cut-off	TP	FP	FN	TN
					H	BBD						
Chen et al.	2001	Maxim	100	70	15	15	70 %	2 score	64	2	6	28
Liu et al.	2002	Boshide	55	30	10	15	55 %	25 %	28	1	2	24
Guo et al.	2007	Zhongshan	69	49	–	20	71 %	5 %	27	5	22	15
Li et al.	2008	Maxim	65	35	15	15	54	0 %	19	2	16	28
Yang et al.	2008	Boshide	56	36	–	20	64	5 %	26	0	10	20
Hu et al.	2009	Santa cruz	107	87	–	20	81	3 score	45	4	42	16
Deng et al.	2013	Maixin	110	90	–	20	82	3 score	59	5	31	15
Zhu et al.	2014	Zhongshan	89	66	–	23	74	5 %	56	2	10	21

**Table 2:** Summary characteristics of 5 studies

Author	Year	Case of stage 1		Case of stage 2		Case of stage 3		Case of control	
		TP	FN	TP	FN	TP	FN	FP	TN
Chen et al.	2001	2	1	59	20	3	0	2	28
Liu et al.	2002	4	1	20	0	1	1	1	24
Li et al.	2008	2	7	9	6	8	3	2	28
Hu et al.	2009	20	22	12	13	13	7	4	16
Zhu et al.	2014	–	–	38	7	18	3	2	10

### Diagnostic accuracy of CD44V6 in discriminating OS from healthy controls or BBD

**Fig. 2:**
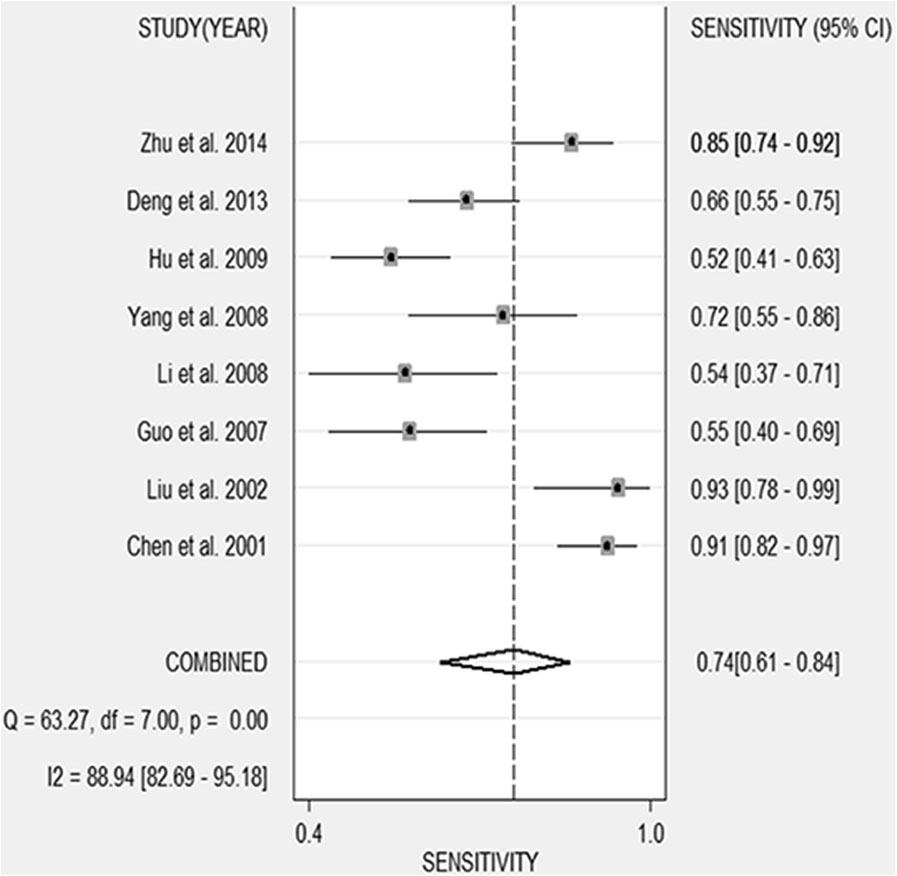
Sensitivity of CD44V6 assay for osteosarcoma

**Fig. 3:**
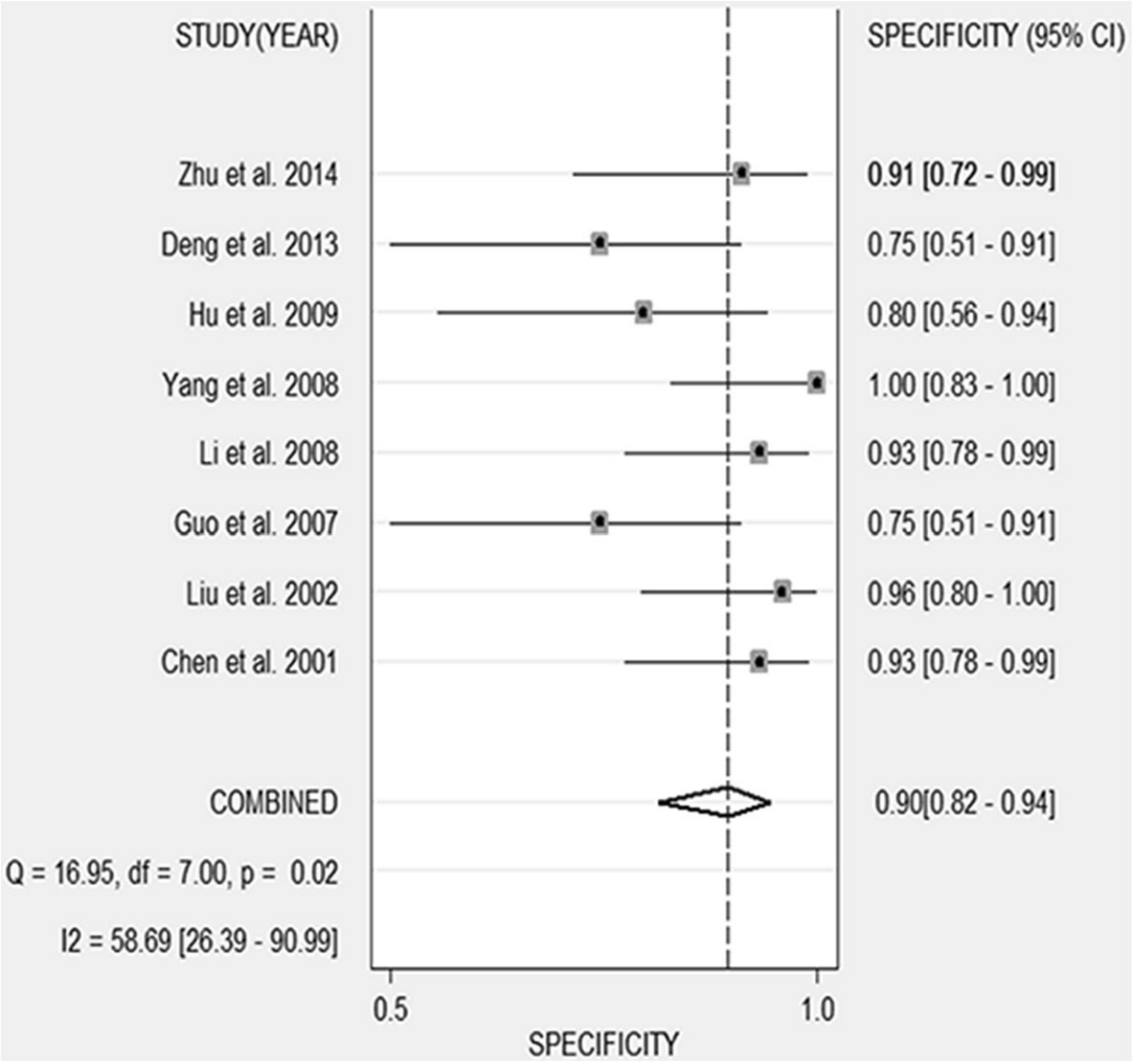
Specificity of CD44V6 assay for osteosarcoma

**Fig. 4:**
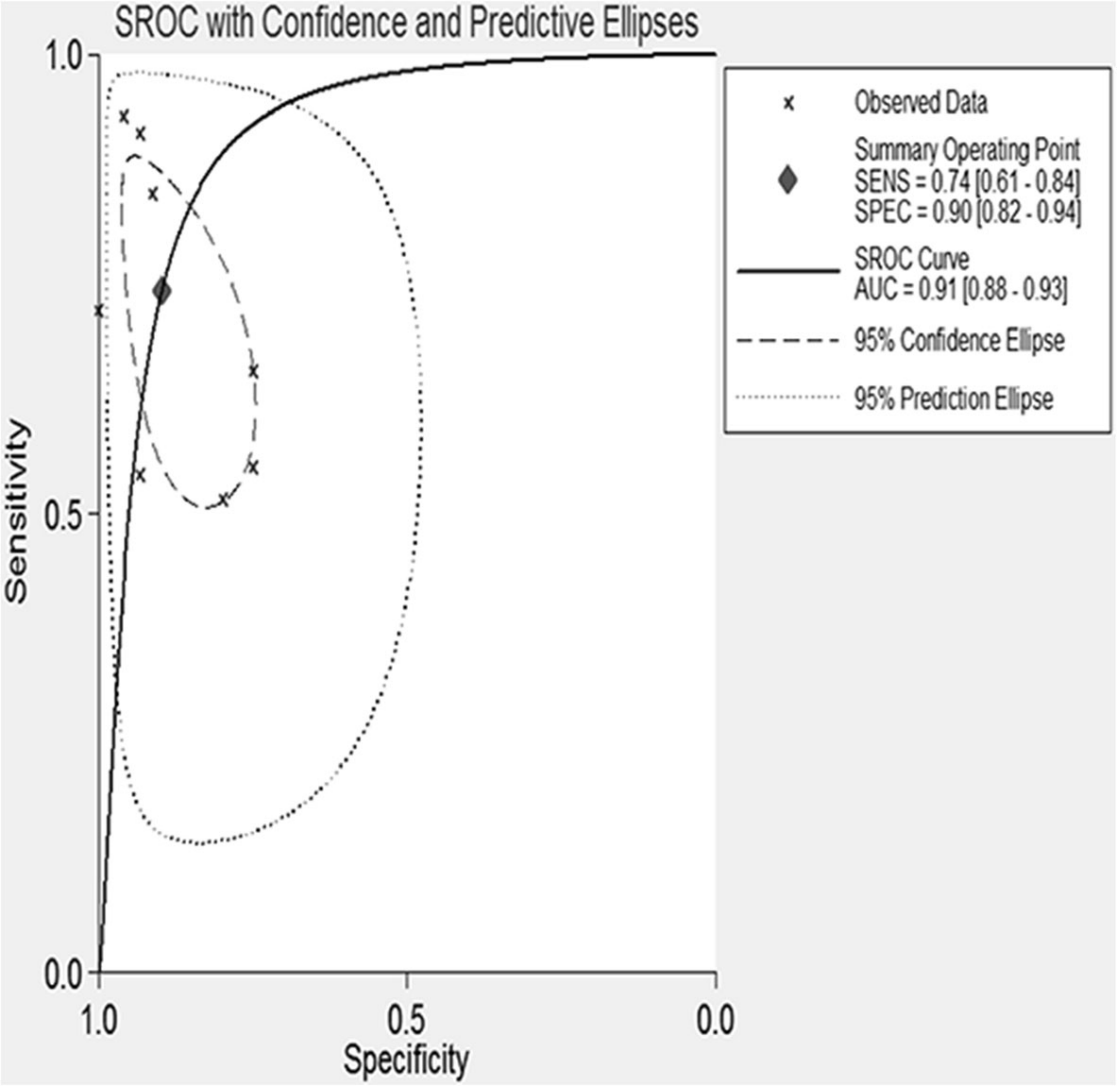
Summary receiver operating characteristic curve

Table 1 shows the accuracy of the CD44V6 in discriminating OS from healthy controls or benign bone disease (BBD). A total of 8 studies involving 651 participants (463 OS patients, 40 healthy controls, and 148 BBD controls) were included in the pooled analysis. The pooled sensitivity was 0.743 (95 % CI 0.606–0.844) (Fig. 2), and pooled specificity was 0.897 (95 % CI 0.818–0.945) (Fig. 3). The AUC was 0.91 (95 % CI 0.88–0.93), and the diagnostic odds ratio (DOR) was 25.267 (95 % CI 8.029–79.574) (Fig. 4). Pooled negative likelihood ratio (NLR) was 0.286 (95 % CI 0.172–0.477), and pooled positive likelihood ratio (PLR) was 7.239 (95 % CI 3.586–14.611). We also tested the diagnostic accuracy of CD44V6 as a biomarker for OS at different stages, as it is shown in Table 3, the diagnostic sensitivity of CD44V6 was increasing with the malignancy of OS from 47 to 75 %, while the diagnostic specificity has no changes (91 %).

**Fig. 5:**
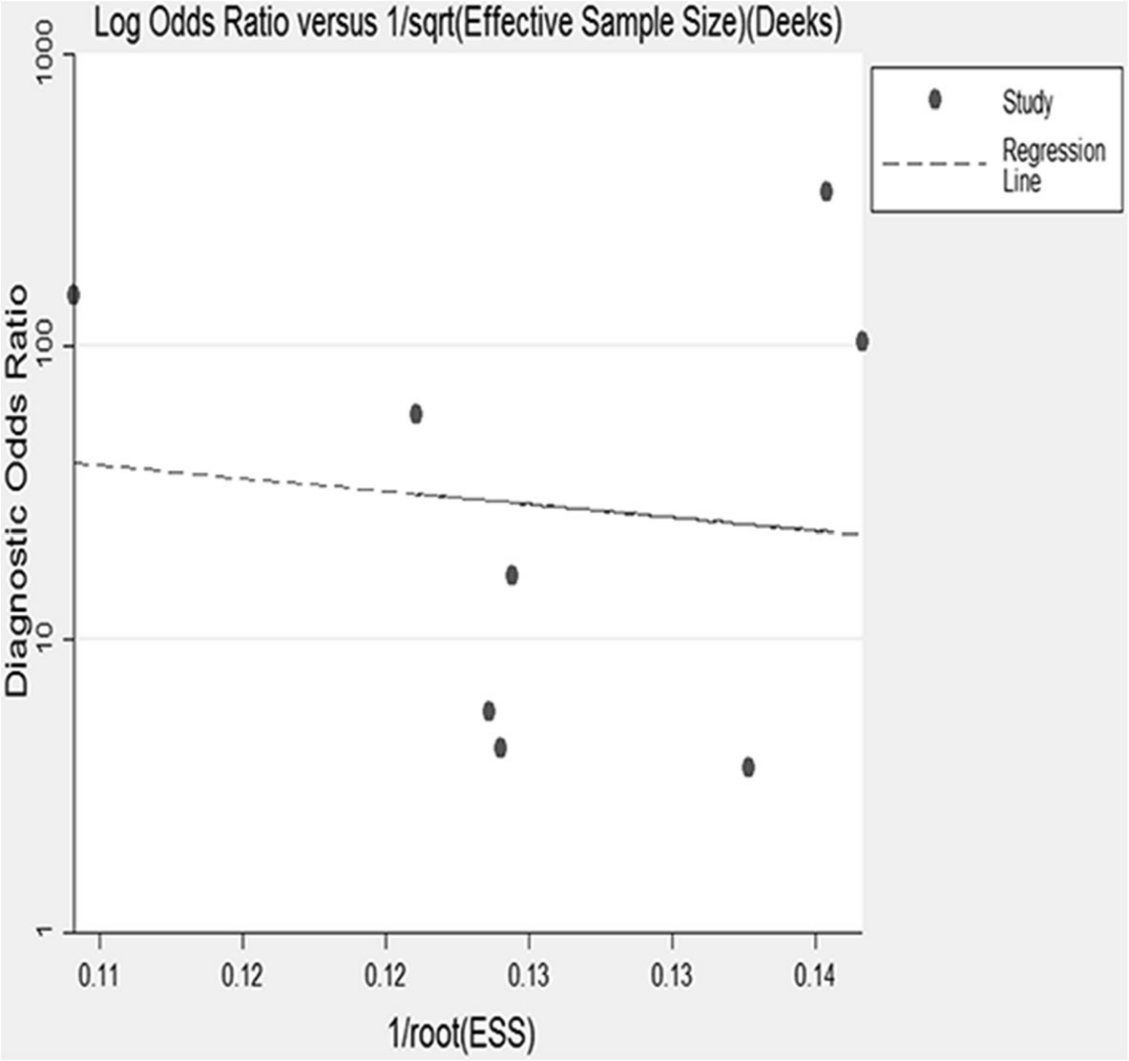
Deeks’ regression test of publication bias

**Table 3:** Summary diagnostic accuracy of CD44V6 for osteosarcoma in different clinical stages

Stage	Number of studies	Case of OS	Case of control	Pooled sensitivity (95 % CI)	Pooled specificity (95 % CI)	AUC (Q)
Stage 1	4	77	105	0.47 (0.34–0.41)	0.91 (0.84–0.96)	0.91 (0.84)
Stage 2	5	184	118	0.75 (0.68–0.81)	0.91 (0.84–0.95)	0.95 (0.87)
Stage 3	5	57	118	0.75 (0.62–0.86)	0.91 (0.84–0.95)	0.89 (0.82)

### Heterogeneity analysis

Substantial heterogeneity was detected among those studies by *I* ^2^ (*I* ^2^ = 90 %, *p* = 0.000). Generally, when *I* ^2^ > 50%, it is considered as heterogeneity. So, we used the random effect model to calculate combined effect indicators. Sensitivity analysis was conducted to investigate the influence of any single study. No significant difference was found after remove of any single study, suggesting that the conclusions are stable.

### Publication bias

Publication bias was not found by Deeks’ regression test (*p* = 0.795) (Fig. 5). The shapes of the funnel plots did not reveal any evidence of obvious asymmetry. The Spearman correlation coefficient indicated that there is no heterogeneity from threshold effects (−0.506, *p* = 0.201).

### Possible sources of heterogeneity and subgroup analysis

Next, meta-regression analyses were then used to identify the sources of heterogeneity. Cut-off values, control groups, and assay kits were considered as the reasonable factors of heterogeneity. After meta-regression analysis, we found neither the cut-off values nor the control groups was the source of heterogeneity (Tables 4 and 5), *p* value for cut-off values was 0.139 (STATA) or 0.492 (Meta-DiSc); *p* value for control groups (STATA) was 0.646 or 0.186 (Meta-DiSc), respectively. Five sources of assay kits (Santa Cruz diagnostics, Maxim, Boshide, Zhongshan, and MAIXIN) were used in 8 studies, and the number of subgroups is not enough to be conducted with meta-regression analysis. Further studies are warranted to confirm whether assay kits are the sources of heterogeneity.

**Table 4:** Univariable bivariate mixed-effects binary meta-regression

Subgroup	MIDAS (*p* value)	Meta-DiSc	
		RDOR	*p* value
Cut-off value	0.139	2.67	0.492
Control group	0.646	0.14	0.186

**Table 5:** Summary data of subgroup analysis

Subgroup	Number of studies	Case of OS	Case of control	Pooled sensitivity (95 % CI)	Pooled specificity (95 % CI)	AUC (Q)
Cut-off value						
Percentage	5	216	118	0.72 (0.66–0.78)	0.92 (0.85–0.96)	0.93 (0.86)
Score	3	247	70	0.68 (0.62–0.74)	0.84 (0.74–0.92)	0.95 (0.90)
Control						
Health	3	135	40	0.82 (0.75–0.88)	0.94 (0.87–0.98)	0.98 (0.94)
BBD	5	328	148	0.65 (0.60–0.70)	0.84 (0.76–0.91)	0.74 (0.68)

### CD44V6 may be used as a diagnostic marker for OS

**Fig. 6:**
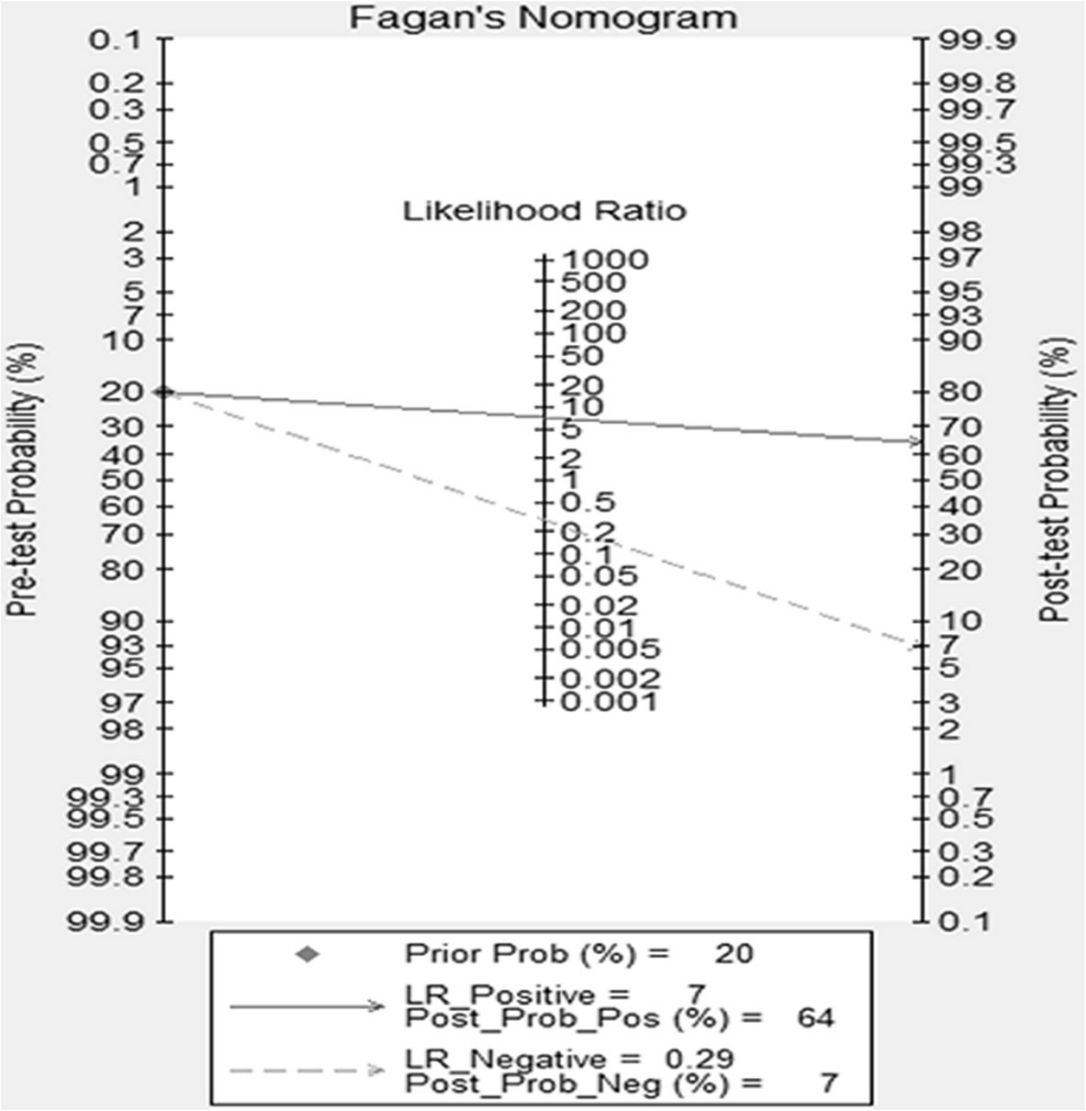
Fagan nomogram of the CD44V6 test for diagnosis of osteosarcoma

Our data shows the positive likelihood ratio is 7.239, which means the OS patients are 7.239 times to have CD44V6 positive result than controls. In order to describe Fagan plot result (Fig. 6), the pre-test probability and the post-test probability were linked by a straight line crossing the likelihood ratio. When 20 % was chosen as the pre-test probability, the post-test probability for CD44V6 positive result is 64 % as a result. Similarly, the post-test probability for CD44V6 negative result was reduced to 7 % with the negative likelihood ratio of 0.29. In conclusion, CD44V6 may be used as a diagnostic maker for OS.

## Discussion

CD44, one of the members of cell adhesion molecules, control cell behavior by mediating contact between cells and the extracellular matrix and are therefore involved in pathological conditions including tumor progression and metastasis [27, 28]. As one of the variant forms of CD44, CD44V6 has been noted to associate with cell adhesion, proliferation, differentiation, and survival and are thereby prone to be involved in tumor progression. For example, CD44V6 has been shown to regulate cell proliferation through MAPK signaling pathway [26]. Additionally, CD44V6 could also suppress tumor apoptosis and facilitate tumor progression through PI3K/Akt signaling pathway. Nakajima and association discovered that CD44V6 may act as an onco-protein in the metastasized OS [29]. Considering that CD44V6 is conspicuous and homogeneous expression within malignant tumors, antibodies recognizing CD44V6 were used in clinical trials for patients suffering from head and neck squamous cell carcinoma (HNSCC). Although the phase I clinical trials looked promising, the studies were abruptly withdrawn after the death of a patient due to skin-related toxicities [30]. Despite the termination of the trials, the onco-genetic role of CD44V6 certainly correlates with aggressive stages of various human cancers. According to the published data, high levels of CD44V6 have been detected in most kinds of carcinomas, such as prostate cancer [31], non-Hodgkin’s lymphomas [32], ovarian cancer [33], cervical cancer [34], and OS [35]. As a result of electronic database search, increasing evidence has showed that high expression of CD44V6 was suggested to be associated with OS. But the small sample size is the limitation of all involved articles. To further explore the relationship between CD44V6 expression levels with OS, the present meta-analysis was conducted to determine whether CD44V6 can be used as a putative diagnostic biomarker for OS.

Eight literatures including 463 cases and 188 controls have been combined in the present meta-analysis. Overall, the results showed SEN of 0.743 (95 % CI 0.606–0.844) and SPE of 0.897 (95 % CI 0.818–0.945), while PLR of 7.239 (95 % CI 3.586–14.611) NLR of 0.286 (95 % CI 0.172–0.477), respectively. The DOR combined the pooled sensitivity and specificity was 25.267, which means CD44V6 could be a promising biomarker in the diagnosis of OS. We then calculated the AUC to assess the diagnostic accuracy of CD44V6 in discriminating OS from controls. An AUC of CD44V6 to OS is 0.91 means a good diagnostic accuracy. The diagnostic value of CD44V6 for OS in different clinical stages was also analyzed. Although pooled specificity is 91 % despite the pathological stage, pooled sensitivity of CD44V6 was increasing with the malignancy of OS from 47 to 75 %.

Heterogeneity has significant impact on interpreting the results from the meta-analysis. After analyzed with the Deeks’ regression test and Spearman correlation coefficient, no evidence of publication bias was detected. Additionally, sensitivity analysis suggests that the diagnostic parameters do not overly rely on one study, which confirmed the robustness of this analysis. Therefore, the source of heterogeneity was then explored by meta-regression. From the data which extracted from the included papers, the cut-off values, control groups and assay kits, were selected to investigate the sources of heterogeneity. After meta-regression analysis, the results show that neither cut-off values nor the control groups are the main source of heterogeneity. Five sources of assay kits were used in 8 studies, and the reagent grouped number is not enough to be performed by meta-regression analysis.

To our knowledge, this is the first meta-analysis and systematic review in summarizing the values of CD44V6 in OS diagnosis. Although our results revealed that CD44V6 was an ideal diagnostic marker of OS, we should prudentially make the conclusion of the association with CD44V6 and OS for some potential limitations. First, because the rarity of primary malignant tumors of the bone, accounting for approximately 0.2 % of all malignancies, the numbers of articles involved in our analysis were relative small, which may weaken the reliability of our results. In future, multicenter trials with larger sample size might need to confirm our results and explore potential factors that may influence diagnostic accuracy. Second, prominent heterogeneity maybe contaminate our analysis results. The heterogeneity was probably due to the cut-off values, control groups, assay kits and others. Under this condition, we try to weaken their effects by using a random effect model.

In summary, despite some limitations mentioned above, our meta-analysis indicated that the elevated CD44V6 expression is significantly associated with OS patients. More clinical studies with larger sample size should be carried out before CD44V6 could be applied to a diagnostic marker in the routine clinical guidance of OS.

## Conclusions

In conclusion, the present results suggest that promoted CD44V6 expression levels are associated with OS and CD44V6 may be used as a diagnostic marker for OS.

## Abbreviations

ALP: Alkaline phosphatase
AUC: Area under the SROC curve
BBD: Benign bone tumor
CD44V6: CD44 variant isoform V6
CNKI: China National Knowledge Internet
DOR: Diagnostic odds ratio
IHC: Immunohistochemistry
NLR: Pooled negative likelihood ratio
NLR: Negative likelihood ratio
OS: Osteosarcoma
PLR: Positive likelihood ratio
PLR: Pooled positive likelihood ratio
QUADAS: Quality Assessment of Diagnostic Accuracy Studies checklist
SEN: Pooled sensitivity
SPE: Pooled specificity
SROC: Summary receiver operating characteristic curve
